# Factors associated with motor complications in Parkinson's disease

**DOI:** 10.1002/brb3.837

**Published:** 2017-09-25

**Authors:** Liis Kadastik‐Eerme, Nele Taba, Toomas Asser, Pille Taba

**Affiliations:** ^1^ Department of Neurology and Neurosurgery University of Tartu Tartu Estonia; ^2^ Estonian Genome Centre University of Tartu Tartu Estonia

**Keywords:** Dyskinesias, levodopa, motor complications, motor fluctuations, Parkinson's disease

## Abstract

**Objectives:**

Levodopa is the most effective therapy for treating Parkinson's disease (PD); however, side effects such as dyskinesias and motor fluctuations may occur after some years of its usage. The aims of this study were to assess the frequency of and factors associated with motor complications among PD patients on levodopa treatment.

**Methods:**

In a cross‐sectional study carried out in 2010–2013, clinical data and treatment details were collected. Logistic regression expressed by odd ratios (OR) and 95% confidence intervals (CI) was conducted to examine the effects of several independent variables on the occurrence of motor complications.

**Results:**

A total of 455 patients were enrolled, among whom 374 were on levodopa. Analysis was performed in 328 patients whose exact duration of levodopa treatment was known. Among patients included in the analysis, 25.9% experienced motor complications; of these, 21% had dyskinesias and 20.1% had motor fluctuations. Based on logistic regression, statistically significant factors associated with the occurrence of motor complications were younger age at onset of the disease, higher levodopa equivalent daily dose (LEDD), shorter time to levodopa initiation, and akinetic‐rigid dominant phenotype of PD.

**Conclusions:**

This study suggests that postponing the start of levodopa therapy and maintaining low daily doses of levodopa might reduce the risk of motor complications. Our results confirm that due to higher risk of motor complications, effectively treating patients with akinetic‐rigid dominant phenotype of PD might be more challenging than for patients whose dominant symptom is tremor.

## 1 INTRODUCTION

Levodopa, the first drug introduced specifically for Parkinson's disease (PD) in 1967 (Lees, Tolosa, & Olanow, 2015), is still the most effective and most frequently prescribed antiparkinsonian medication. As the disease progresses, however, levodopa becomes less effective, and larger doses are needed to achieve the same effect, increasing the risk of unwanted side effects and motor complications (Aquino & Fox, 2015; Vijayakumar & Jankovic, 2016). Dyskinesias are involuntary movements, usually affecting face and limbs, most typically presenting as peak dose or biphasic. Off‐period dystonia is another dyskinesia type that frequently involves the foot and leg; it is often painful and usually starts at night or early in the morning. Motor fluctuations are alterations of clinical state with “off”‐periods manifesting in worsening of motor symptoms (Aquino & Fox, 2015; Vijayakumar & Jankovic, 2016).

Based on the findings of various observational surveys using different study designs, the frequency of dyskinesias ranges from 26% to 44% and that of motor fluctuations from 22% to 64% (Nicoletti et al., 2016; Yoritaka et al., 2013; Martínez‐Martín et al., 2014; Hashim et al., 2014; Larsen, Karlsen, & Tandberg, 2000; Schrag & Quinn, 2000). Several studies have shown the prevalence of motor complications to increase with the duration of the disease (Nicoletti et al., 2016; Hashim et al., 2014; Schrag & Quinn, 2000; Scott, Macleod, & Counsell, 2016; García‐Ruiz, Del Val, Fernández, & Herranz, 2012; Stocchi et al., 2014). Motor complications may affect more than half of patients within 5 years of diagnosis (Bjornestad et al., 2016) and by the end of the first decade, up to 90% of patients may exhibit dyskinesia and 60% motor fluctuations (García‐Ruiz et al., 2012). One of the longest follow‐up studies demonstrated that by the end of the second decade, motor complications were present in 100% of patients who were on at least 300 mg levodopa per day (Hely, Reid, Adena, Halliday, & Morris, 2008). Risk of development of motor complications may also rise in a dose‐dependent manner (Schrag & Quinn, 2000; Scott et al., 2016; Warren Olanow et al., 2013) but also in a levodopa duration‐dependent manner (Hashim et al., 2014; Schrag & Quinn, 2000).

Other variables that potentially increase the risk for motor complications have been reported: younger age at onset (Hashim et al., 2014; García‐Ruiz et al., 2012; Bjornestad et al., 2016; Warren Olanow et al., 2013), female gender (Yoritaka et al., 2013; Larsen et al., 2000; Schrag & Quinn, 2000; Scott et al., 2016; Bjornestad et al., 2016), initial treatment with levodopa (Schrag & Quinn, 2000; García‐Ruiz et al., 2012), and a higher score on the Unified Parkinson's Disease Rating Scale (UPDRS) Part II (Stocchi et al., 2014; Warren Olanow et al., 2013). Additionally, a low body weight (Warren Olanow et al., 2013) presence of tremor at diagnosis (Scott et al., 2016), higher levodopa equivalent dose, (Nicoletti et al., 2016) and higher MMSE score (Scott et al., 2016) have been shown to predict a higher rate of dyskinesias, while younger age (Stocchi et al., 2014; Bjornestad et al., 2016) and UPDRS Part III score (Bjornestad et al., 2016) predict a higher rate of motor fluctuations.

The association between the emergence of motor complications and the time from the first symptoms of PD until levodopa initiation has been the subject of debate. There is growing evidence suggesting that delaying the start of levodopa therapy might not be associated with smaller risk of motor complications in the long term (Scott et al., 2016; Cilia et al., 2014). The relationship between motor complications and distinct clinical motor phenotypes of PD has not been studied enough. There is some evidence that tremor‐dominant subtype may indicate a lower probability of motor complications (Nicoletti et al., 2016. To extend the understanding between motor complications and a range of clinical factors, the current study was carried out.

The objectives of this paper were (1) to describe the frequency of motor complications among patients with PD who receive levodopa treatment and (2) to assess the effect of various demographic and clinical factors on the occurrence of motor complications.

## 2 PATIENTS AND METHODS

### 2.1 Study design and patients

A cross‐sectional observational study was conducted in three medical centers in different regions of Estonia (Tartu, Pärnu, and Tallinn) between 2010 and 2013. As it was primarily an epidemiological study, all the patients in the region were included, representing different stages and phenotypes of the disease. PD was diagnosed based on the diagnostic criteria of the Queen Square Brain Bank Criteria (Gibb & Lees, 1988; Lees, Hardy, & Revesz, 2009). No specific exclusion criteria were set. From the initial cohort of 486 parkinsonian patients, 16 were excluded due to correction of diagnoses including other tremors, secondary or atypical parkinsonism, or Lewy body dementia, and 15 more were excluded due to refusal or incomplete testing. A total of 455 patients were included in the final analysis.

Information from all available sources was obtained for the case ascertainment. A majority of the study participants were outpatients (*n *=* *390). A few were inpatients (*n *=* *14), in nursing homes (*n *=* *23) or visited at their own homes (*n *=* *28). Ethical approval was obtained from the Research Ethics Committee of the University of Tartu. All patients provided signed informed consent.

### 2.2 Data collection and outcome measures

A semistructured interview based on a special case report form containing items on demographic and clinical data was performed, along with a neurological examination. Total levodopa equivalent daily dose (LEDD) was calculated using the standardized conversion formula (Tomlinson et al., 2010). The data on the prevalence of motor complications was obtained from two sources: patient cards and Part IV of the MDS‐UPDRS (Goetz et al., 2008). Patient cards were provided with binary questions assessing motor fluctuations, dyskinesias, and off‐dystonia that had occurred at any time during the disease. All items in the MDS‐UPDRS are scored on a scale of 0 to 4 (normal/mild/slight/moderate/severe). The presence of motor complications over the past week was indicated by a score of ≥1 on MDS‐UPDRS items 4.1 and 4.6 (off‐dystonia was included among dyskinesias) and item 4.3 (motor fluctuations). All other parts of the MDS‐UPDRS were also performed: Part I—nonmotor aspects; Part II—motor experiences of daily living; and Part III—motor examination.

During the neurological examination, motor phenotype was determined based on the most prevalent symptom: the dominance of tremor with other motor symptoms of only a mild level; nontremor‐dominant PD which includes syndromes with akinesia and rigidity; or postural instability and gait disorder (PIGD). The clinical stage was assessed using the Hoehn and Yahr scale (HY) (Hoehn & Yahr, 1967). To measure depression, the Beck Depression Inventory (BDI) was performed (Beck, Ward, Mendelson, Mock, & Erbaugh, 1961). For the screening of cognitive impairment, the Mini Mental State Examination (MMSE) was performed (Folstein, Folstein, & McHugh, 1975). For the purpose of evaluation of quality of life, the Parkinson's Disease Quality of Life Questionnaire (PDQ‐39) was used (Peto, Jenkinson, Fitzpatrick, & Greenhall, 1995).

### 2.3 Statistical analysis

Primary analysis involved the evaluation of the frequency of motor complications in our sample of PD patients who received levodopa therapy. Although, 374 patients received levodopa therapy at the time of examination, 28 had started with levodopa the same day or a few days before the examination; therefore, they were excluded from the analysis. Another 18 levodopa users were excluded from the frequency analysis of motor complications due to missing the variable indicating the duration of their levodopa therapy.

Secondary analysis comprised descriptive statistics for the total study sample (*n* = 328), for the patients on levodopa who did not have motor complications (*n* = 243), and for the patients on levodopa who had motor complications (*n* = 85). Group comparisons were performed using the two‐sample *t*‐test, chi‐squared test, Fischer's exact test, and Mann–Whitney *U* test. Multiple comparisons were corrected with the Bonferroni method.

Tertiary analysis incorporated logistic regression models to examine the effects of multiple predefined variables on the binary outcome variable (whether motor complications were present). Based on the literature and clinical experience, the following variables were entered into the main model: age, gender, age at onset of PD, time from initial symptoms until starting levodopa, LEDD, MDS‐UPDRS Parts II and III scores, BDI, PDQ‐39 SI score, and akinetic‐rigid dominant, tremor‐dominant and PIGD‐dominant motor phenotype of the disease. Odds ratios (OR) with 95% confidence intervals (CI) were calculated for all abovementioned variables. The results were considered statistically significant at the *p* < .05 level. Statistical analysis was performed in R version 3.3.1.

## 3 RESULTS

### 3.1 Prevalence of motor complications

Out of 328 patients on levodopa (130 men and 198 women), some type of motor complication was present in 85 patients (25.9%). Dyskinesias were present in 21% of patients and motor fluctuations in 20.1%, while 61% of all of those with motor complications experienced both fluctuations and dyskinesias. The sources for the case identification of 69 patients with dyskinesias were distributed as follows: 61% were recorded on both patient cards and MDS‐UPDRS Part IV, 29% on patient cards, and 10% on MDS‐UPDRS Part IV. Presence of motor fluctuations among 66 patients was indicated as follows: 68% from both patient cards and MDS‐UPDRS Part IV, 20% from MDS‐UPDRS Part IV, and 12% from patient cards. The functional impact of dyskinesias was reported to be at least moderate (MDS‐UPDRS item 4.2 ≥ 3) by 17.4% of patients. Just over one‐third of those patients with motor fluctuations indicated that the functional impact of off‐state was at least moderate (MDS‐UPDRS item 4.4 ≥ 3). As Table 1 shows, prevalence of motor complications was relatively low, earlier in the course of PD, but increased with the duration of levodopa treatment.

**Table 1:** Motor complications in levodopa users according to the duration of levodopa treatment

Patients on levodopa therapy	Motor fluctuations	Dyskinesias	Motor fluctuations and/or dyskinesias
All patients (*n* = 328)	66 (20.1%)	69 (21%)	85 (25.9%)
≤2.5 years (*n* = 147)	7 (4.8%)	5 (3.4%)	9 (6.1%)
2.6‐5 years (*n* = 66)	14 (21.2%)	11 (16.7%)	19 (28.8%)
5.1‐10 years (*n* = 62)	19 (30.6%)	24 (38.7%)	27 (43.5%)
10.1‐15 years (*n* = 38)	15 (39.5%)	19 (50%)	19 (50%)
≥15.1 years (*n* = 15)	11 (73.3%)	10 (66.7%)	11 (73.3%)
*p*‐value^a^	<.0001	<.0001	<.0001

a Fisher's exact test was used to examine the significance of the association (contingency) between the two kinds of classification.

Statistically significant *p*‐values were set at .017.

### 3.2 Characteristics of patients

The clinical profile of the entire study sample, as well as the group comparisons of those with and without motor complications, is depicted in Table 2. The ratio of females to males was similar in patients with and without motor complications (approximately 60% of females and 40% of males in both groups). Patients with motor complications were younger than patients without motor complications (71.7 ± 8.9 vs. 75.2 ± 8.2 years; *t*‐test, *p* = .002). The differences were most evident when regarding age at PD onset and duration of the disease. The outcomes of most of the clinometric scales differed more or less between patients with and without motor complications. No differences were seen in the severity of PD assessed by HY, with a median value of 3 in both groups. No statistically significant differences were found in the MMSE scores, with the median value of 27 among those without and 28 among those with motor complications (Mann–Whitney *U* test, *p* = .08). Duration of levodopa treatment was higher among patients with motor complications compared to those without (8.8 ± 5.3 vs. 3.7 ± 4.1 years; Mann–Whitney *U* test, *p* < .0001). Other antiparkinsonian treatment‐related characteristics are shown in Table 3.

**Table 2:** Disease‐related characteristics of PD patients with and without motor complications

Variable	All patients *n* = 328	Motor complications		*p*‐value
		without *n* = 243	with *n* = 85	
Age at onset, years				
Mean (*SD*)	66.4 (10.4)	68.6 (9.7)	60 (9.6)	<.0001^a^
Median (ranges)	67 (35‐91)	70 (35‐91)	61 (35‐83)	
Missing	9	6	3	
Disease duration, years				
Mean (*SD*)	7.8 (6.2)	6.5 (5.8)	11.5 (5.7)	<.0001^b^
Median (ranges)	6 (0‐43)	5 (0.3‐43)	10.6 (2.8‐35)	
Missing	9	9	3	
Time from PD onset until diagnosis, years				
Mean (*SD*)	1.8 (3.0)	1.9 (3.4)	1.4 (1.7)	.484^b^
Median (ranges)	1 (0‐10)	1 (0‐10)	1 (0‐10)	
Missing	12	9	3	
Clinical subtype of PD, *n* (%)				
Tremor	122 (37.2)	103 (42.4)	19 (22.4)	.002^c^
Akinetic‐rigid	120 (36.6)	77 (31.7)	43 (50.6)	
PIGD	86 (26.2)	63 (25.9)	23 (27.1)	
BDI				
Mean (*SD*)	15.7 (9.0)	15.1 (8.5)	17.4 (10.2)	.126^b^
Median (ranges)	14 (0‐48)	13 (0‐45)	15 (2‐48)	
Missing	44	32	12	
PDQ‐39 SI				
Mean (*SD*)	31.9 (15.9)	30.5 (16.1)	35.9 (14.8)	.011^a^
Median (ranges)	29.9 (1.6‐71.1)	27.4 (1.6‐71.1)	35.1 (6.6‐68.6)	
Missing	43	31	12	
MDS‐UPDRS Part I				
Mean (*SD*)	12.8 (6.8)	12.0 (6.5)	14.8 (7.1)	.002^a^
Median (ranges)	12 (0‐38)	12 (0‐35)	14 (1‐38)	
MDS‐UPDRS Part II				
Mean (*SD*)	17.8 (8.9)	16.4 (8.1)	21.8 (10.0)	<.0001^a^
Median (ranges)	17 (0‐45)	15 (1‐45)	20 (3‐42)	
MDS‐UPDRS Part III				
Mean (*SD*)	44.1 (18.0)	42.3 (16.6)	49.1 (20.7)	.007^a^
Median (ranges)	41.5 (9‐106)	40 (9‐83)	48 (11‐106)	

PD, Parkinson's disease; *SD*, standard deviation; PIGD, postural instability, and gait disorder; BDI, the Beck Depression Inventory; PDQ‐39 SI, the Parkinson's Disease Questionnaire Summary Index; MDS‐UPDRS, the Movement Disorders Society's Unified Parkinson's Disease Rating Scale.

a *t*‐test.

b Mann–Whitney *U* test.

c chi‐squared test.

Statistically significant *p*‐values after Bonferroni's correction were set at .0045.

**Table 3:** Antiparkinsonian treatment of PD patients with and without motor complications

Variable	All patients *n* = 328	Motor complications		*p*‐value
		without *n* = 243	with *n* = 85	
Current antiparkinsonian treatment, *n* (%)				
Levodopa summarily	328 (100%)	243 (100%)	85 (100%)	
Regular levodopa	62 (18.9%)	50 (20.6%)	12 (14.1%)	.251^a^
CR levodopa	220 (67.1%)	181 (74.5%)	39 (45.9%)	<.0001^a^
Soluble levodopa	6 (1.8%)	2 (0.8%)	4 (4.7%)	.041^b^
Levodopa +	63 (19.2%)	19 (7.8%)	44 (51.8%)	<.0001^a^
COMT inhibitors				
Dopamine agonists	103 (31.4%)	55 (22.6%)	48 (56.5%)	<.0001^a^
MAO‐B inhibitors	64 (19.5%)	46 (18.9%)	18 (21.2%)	.771^a^
Amantadine	67 (20.4%)	32 (13.2%)	35 (41.2%)	<.0001^a^
Anticholinergics	9 (27%)	8 (3.3%)	1 (1.2%)	.456^b^
Time from PD onset until levodopa treatment, years				
Mean (*SD*)	2.6 (3.6)	2.6 (3.9)	2.5 (2.7)	.629^c^
Median (ranges)	2 (0‐10)	2 (0‐38)	2 (0‐13)	
Missing	9	6	3	
Levodopa daily dose, mg				
Mean (*SD*)	433.5 (221.9)	390.8 (184.0)	555.3 (271.8)	<.0001^c^
Median (ranges)	400 (100‐1300)	400 (100‐1000)	500 (100‐1300)	
LEDD, mg				
Mean (*SD*)	537.1 (362.7)	421.7 (222.9)	865.8 (469.3)	<.0001^c^
Median (ranges)	450 (75‐2518)	399 (75‐1120)	808.5 (100‐2518)	
Missing	1	1	0	

PD, Parkinson's disease; CR, controlled release; *SD*, standard deviation; LEDD, levodopa equivalent daily dose.

a chi‐squared test.

b Fischer's exact test.

c Mann–Whitney *U* test.

Statistically significant *p*‐values after Bonferroni's correction were set at .0041.

### 3.3 Logistic regression analysis

The results of the logistic regression can be found in Table 4. An increase of LEDD by one unit (keeping all other variables fixed) increased the odds of having motor complications by 0.3% (*p* = .0005). As a hypothetical example, if there were two patients who differed in that one of them had a LEDD of 1,000 mg and the other 1,500 mg, and they were identical in all other variables entered into the model, then based on our model, the second patient would have 1.002561^500^ = 3.59 times higher odds than the first of having motor complications.

**Table 4:** Factors associated with motor complications in PD: the logistic regression model

Variable	Odds ratio	95% CI
Gender	1.616	0.721–3.727
Age	1.106*	1.018–1.203
Age at onset of PD	0.837**	0.766–0.910
Time from onset to initiation with levodopa	0.746**	0.624–0.870
Akinetic‐rigid subtype	2.559*	1.087–6.199
PIGD subtype	1.024	0.379–3.188
LEDD	1.003**	1.001–1.004
MDS‐UPDRS II	1.058	0.979–1.145
MDS‐UPDRS III	0.998	0.969–1.027
BDI	1.008	0.956–1.063
PDQ‐39 SI	0.987	0.956–1.019

PD, Parkinson's disease; CI, confidence interval; PIGD, postural instability, and gait disorder; LEDD, levodopa equivalent daily dose; MDS‐UPDRS, the Movement Disorders Society's Unified Parkinson's Disease Rating Scale; BDI, the Beck Depression Inventory; PDQ‐39 SI, the Parkinson's Disease Questionnaire Summary Index.

a **p < .001.

b *p < .05.

Keeping all other variables fixed, each year of earlier initiation of levodopa from PD onset increased the odds of having motor complications by 34% (*p* = .0006); each year younger of onset age increased the odds by 19.5% (*p* = .000045); each year older of age at the time of examination increased the odds by 11% (*p* = .018); and having akinesia‐rigid phenotype of the disease (as opposed to the dominance of tremor) increased the odds by 156% (*p* = .033). No statistically significant associations were found between motor complications and gender (*p* = .3), PIGD‐dominant phenotype of disease as opposed to tremor‐dominant phenotype (*p* = .9), BDI (*p* = .8), MDS‐UPDRS Part II and III scores (*p* = .2, *p* = .9, respectively), or PDQ‐39 scores (*p* = .5).

To confirm that the effect of clinical phenotype and LEDD is not due to possible confounders (duration of PD and duration of levodopa treatment), we conducted an analysis using the second model, in which we adjusted for these two possible confounders. LEDD (OR = 0.003; 95% CI: 1.001 to 1.004, *p* = .0004) and akinesia‐rigid phenotype as opposed to tremor‐dominant phenotype (OR = 2.549; 95% CI: 1.1 to 6.2, *p* = .04) still emerged as significant independent variables associated with the occurrence of motor complications with almost the same OR coefficients as in the first model. As a post hoc analysis, we examined the LEDD of patients stratified by age and found that younger patients tended to be on a higher LEDD than older patients.

In order to assess a possible association between levodopa daily dose and the presence of motor complications, we conducted an analysis based on another logistic model in which we entered all the same variables as in the first model but included levodopa daily dose instead of LEDD. No evidence was found for associations between motor complications and levodopa daily dose (OR = 1.002; 95% CI: 1.0 to 1.003, *p* = .07).

## 4 DISCUSSION

This study aimed to address two main questions. Our first goal was to assess the prevalence of motor complications among a representative sample of PD patients in Estonia. Our second aim was to examine the association between multiple factors and the development of the disabling side effects of levodopa.

With respect to the first research question, we found a slightly lower frequency of motor complications in our patients than several other studies (approximately 20% of our study participants with either of the motor complications vs. more than 22% of motor fluctuations and more than 26% of dyskinesias in other studies) (Nicoletti et al., 2016; Yoritaka et al., 2013; Martínez‐Martín et al., 2014; Hashim et al., 2014; Larsen et al., 2000; Schrag & Quinn, 2000). It is possible that the somewhat lower frequency of motor complications was partly due to the profile of the study participants. Regarding the usage of levodopa, the mean daily levodopa dose of 434 mg in our study was lower than that reported in other studies (500 mg in the study by Hashim et al. and 548 mg in the study by Yoritaka et al.) (Yoritaka et al., 2013; Hashim et al., 2014). As this study was part of an epidemiologic survey of PD, all patients in the region were enrolled, included early cases. Among patients with early stages of PD who have been on levodopa therapy for a short time, motor complications are rather unexpected, a phenomenon demonstrated in Table 1. This finding contrasts with the results of Stocchi et al. (Stocchi et al., 2014); in which more than half of patients under levodopa therapy for 1 to 2 years were diagnosed as having wearing‐off symptoms, whereas only a minority of patients under levodopa ≤2.5 years had motor fluctuations in our study. Discrepancies in results could be caused by the variability in cohorts or by differing methodology of assessment. MDS‐UPDRS Part IV was used in our study, whereas Stocchi et al. used a patient‐assessed 19‐item Wearing‐off Questionnaire with the aim to detect wearing‐off symptoms in early stages of PD (Stocchi et al., 2014).

With respect to the second research question, our findings confirm the results by others showing that motor complications are perhaps the greatest concern for PD patients who receive high cumulative dopaminergic doses (Nicoletti et al., 2016; Yoritaka et al., 2013; Larsen et al., 2000; Schrag & Quinn, 2000; Scott et al., 2016; Warren Olanow et al., 2013). Instead of levodopa daily dose, we chose LEDD as a total daily dose of medication expressing dose intensity of different antiparkinsonian drug regimens. Levodopa equivalent dose of a drug is defined as that which produces the same level of symptomatic control as 100 mg of immediate release levodopa (Tomlinson et al., 2010). Advanced PD patients are often treated with a combination of antiparkinsonian medications to handle their PD symptoms and levodopa‐induced side effects. Therefore, especially in Estonia, where levodopa doses seem to be somewhat lower than in other countries, levodopa daily dose may underestimate the total dopaminergic load. Furthermore, approximately half of patients (57%) with motor complications were on dopamine agonists, which were used more than twice as than they were by patients without motor complications (Mann–Whitney *U*,* p* < .0001). Our findings on LEDD emerged from the multivariate regression analysis as a significant predictor of motor complications that was not found for the levodopa daily dose. It may support our observation that the levodopa dose alone may underestimate the cumulative dopaminergic burden. Although, motor complications are traditionally considered to result from long‐term use of levodopa, (Aquino & Fox, 2015) the total burden of antiparkinsonian treatment may play a significant role in the emergence of motor complications.

One of the key findings of this study is that a shorter duration of disease until the initiation of levodopa appears to increase the odds of motor complications. This finding is supportive of a few studies indicating that early initiation of levodopa might be a risk factor for motor complications (Schrag & Quinn, 2000; Denny & Behari, 1999) but contrary to some other surveys suggesting that delaying the start of levodopa therapy is not associated with a smaller risk of motor complications in the long term (Scott et al., 2016; Cilia et al., 2014). Our results support the opinion that younger age at onset of PD seems to be a risk factor for motor complications per se (Hashim et al., 2014; García‐Ruiz et al., 2012; Bjornestad et al., 2016; Warren Olanow et al., 2013). Post hoc analysis revealed that the younger patients tended to be on a higher LEDD than older patients. They might be more prone to the higher usage of dopaminergic treatment in order to reduce their parkinsonian symptoms than older patients as a result of having more commitments concerning their employment status and families than older patients. Levodopa may be postponed in patients who are not troubled by their PD‐related motor symptoms and in patients with young‐onset PD who are at the highest risk for developing levodopa‐related complications (Jankovic & Poewe, 2012). At the same time, the possibility of the different effects of dyskinesias and motor fluctuations on quality of life should be taken into account. Some studies have found that PD patients with fluctuations have lower quality of life than patients without fluctuations (Stocchi et al., 2014; Skorvanek et al., 2015) but no such association was found between quality of life and dyskinesias in another study (Hechtner et al., 2014). In our survey, a majority of patients with dyskinesias reported that drug‐induced complications impair their functional ability only mildly, and severe dyskinesias were rarely described. This finding may provide some support for the possibility that patients themselves might not be particularly troubled by their dyskinesias, at least when those features are mild. In summary, we conclude that the traditional clinical approach of balancing efficacy and risk of motor complications is needed for each patient, using the lowest dose of levodopa that will provide satisfactory clinical control (Warren Olanow et al., 2013). Still, improved quality of life should be the ultimate goal of our treatments.

In disagreement with the data of recent studies, we did not find female gender to be associated with higher occurrence of motor complications (Yoritaka et al., 2013; Scott et al., 2016; García‐Ruiz et al., 2012; Stocchi et al., 2014; Bjornestad et al., 2016; Warren Olanow et al., 2013). It has also been suggested that the severity of motor PD symptoms predicts a higher risk of developing motor complications (Bjornestad et al., 2016). However, this does not appear to be the case in our study, supporting the results of another study based on a cohort of incident PD patients (Scott et al., 2016). Although, we found more severe MDS‐UPDRS Part II scores for those experiencing motor complications, according to logistic regression analysis, everyday activities were not significantly associated with the presence of levodopa‐induced side effects, which does not support the observation by one other study based on a cohort of early PD patients (Warren Olanow et al., 2013).

One rather important finding was that the akinetic‐rigid phenotype of PD emerged as a significant independent associated factor in the higher occurrence of motor complications. This association remained significant even after including possible confounders, such as duration of PD and levodopa therapy, to the second model. Patients with nontremor‐dominant types of PD are characterized as being more often depressed and having a more severe clinical picture, higher LEDD, and longer disease duration than patients with tremor‐dominant subtype (Burn et al., 2012). In a recent case‐control study in Italy investigating the relationship between clinical phenotype and the risk of developing dyskinesias, a significant negative association between tremor‐dominant phenotype as an initial PD manifestation and levodopa‐induced dyskinesia was established (Nicoletti et al., 2016). Another case‐control study conducted in Finland investigated differences in the binding of striatal dopamine transporter and the extent of caudate dopamine terminal loss, and the consequent dopamine function was relatively more preserved in PD patients with tremor compared to akinetic‐rigid patients (Kaasinen, Kinos, Joutsa, Seppänen, & Noponen, 2014). Our study allows us to confirm information about the clinical heterogeneity of distinct PD phenotypes. Due to the higher risk for motor complications, achieving effective treatment results in patients whose dominant symptoms are akinesia and rigidity might be more challenging than in patients with the tremor‐dominant disease.

Our study has several strengths. First, the case identification in our study was based on all available sources in the area, and we accordingly enrolled a relatively large sample of patients with PD, including early cases as well as both moderately and severely ill patients. Thus, the results may be extrapolated to the Estonian PD population as a whole. Secondly, the evaluation of the participants was thorough, and wide‐ranging aspects (motor, nonmotor, functional, cognitive, and emotional) were assessed based on validated clinometric scales and questionnaires. Therefore, we believe that the measurements are accurate and reflect the profile of study participants correctly.

Some limitations of this study need to be taken account when interpreting the findings. The main weakness of the study is a relatively small sample size of patients with motor complications, which can be a source of low power for the logistic regression analysis. The second limitation might be a potential underestimation of motor complications with subtle presentations, especially among patients with impaired cognitive ability. Finally, due to the cross‐sectional design of our study, each patient was assessed once, so no pattern of progression of the disease could be estimated and causes and effects could not be determined.

In conclusion, the most important findings of this study were that patients receiving a higher LEDD and having akinetic‐rigid dominant phenotype as opposed to tremor‐dominant phenotype of the disease are at greater risk of having motor complications. The results of the research do not support the idea that levodopa should be initiated as early as possible; instead, postponing the start of levodopa, together with prescribing optimal daily doses, might reduce the odds of motor complications.

## CONFLICT OF INTERESTS

The authors declare that they have no financial or non‐financial competing interests.

## References

[brb3837-bib-0001] Aquino, C. C. , & Fox, S. H. (2015). Clinical spectrum of levodopa‐induced complications. Movement Disorders, 30(1), 80–89. 25488260 10.1002/mds.26125

[brb3837-bib-0002] Beck, A. T. , Ward, C. H. , Mendelson, M. , Mock, J. , & Erbaugh, J. (1961). An inventory for measuring depression. Archives of General Psychiatry, 4(6), 561–571. 13688369 10.1001/archpsyc.1961.01710120031004

[brb3837-bib-0003] Bjornestad, A. , Forsaa, E. B. , Pedersen, K. F. , Tysnes, O. B. , Larsen, J. P. , & Alves, G. (2016). Risk and course of motor complications in a population‐based incident Parkinson's disease cohort. Parkinsonism and Related Disorders, 22, 48–53. 26585090 10.1016/j.parkreldis.2015.11.007

[brb3837-bib-0004] Burn, D. J. , Landau, S. , Hindle, J. V. , Samuel, M. , Wilson, K. C. , Hurt, C. S. , … PROMS‐PD Study Group . (2012). Parkinson's disease motor subtypes and mood. Movement Disorders, 27(3), 379–386. 22162098 10.1002/mds.24041

[brb3837-bib-0005] Cilia, R. , Akpalu, A. , Sarfo, F. S. , Cham, M. , Amboni, M. , Cereda, E. , … Pezzoli, G. (2014). The modern pre‐levodopa era of Parkinson's disease: Insights into motor complications from sub‐Saharan Africa. Brain, 137(10), 2731–2742. 25034897 10.1093/brain/awu195 PMC4163032

[brb3837-bib-0006] Denny, A. P. , & Behari, M. (1999). Motor fluctuations in Parkinson' s disease. Journal of the Neurological Sciences, 165(1), 18–23. 10426141 10.1016/s0022-510x(99)00052-0

[brb3837-bib-0007] Folstein, M. F. , Folstein, S. E. , & McHugh, P. R. (1975). “Mini‐mental state”. A practical method for grading the cognitive state of patients for the clinician. Journal of Psychiatric Research, 12(3), 189–198. 1202204 10.1016/0022-3956(75)90026-6

[brb3837-bib-0008] García‐Ruiz, P. J. , Del Val, J. , Fernández, I. M. , & Herranz, A. (2012). What Factors Influence Motor Complications in Parkinson Disease?: A 10‐Year Prospective Study. Clinical Neuropharmacology, 35(1), 1–5. 22139623 10.1097/WNF.0b013e31823dec73

[brb3837-bib-0009] Gibb, W. R. , Lees, A. J. (1988). The relevance of the Lewy body to the pathogenesis of idiopathic Parkinson's disease. Journal of Neurology, Neurosurgery and Psychiatry, 51(6), 745–752. 10.1136/jnnp.51.6.745 PMC1033142 2841426

[brb3837-bib-0010] Goetz, C. G. , Tilley, B. C. , Shaftman, S. R. , Stebbins, G. T. , Fahn, S. , Martinez‐Martin, P. , … Movement Disorder Society UPDRS Revision Task Force . (2008). Movement Disorder Society‐Sponsored Revision of the Unified Parkinson's Disease Rating Scale (MDS‐UPDRS): Scale presentation and clinimetric testing results. Movement Disorders, 23(15), 2129–2170. 19025984 10.1002/mds.22340

[brb3837-bib-0011] Hashim, H. Z. , Norlinah, M. I. , Nafisah, W. Y. , Tan, H. J. , Raymond, A. A. , & Tamil, A. M. (2014). Risk factors and predictors of levodopa‐induced dyskinesia among multiethnic Malaysians with Parkinson's disease. International Journal of Neuroscience, 124(3), 187–191. 23952588 10.3109/00207454.2013.833511

[brb3837-bib-0012] Hechtner, M. C. , Vogt, T. , Zöllner, Y. , Schröder, S. , Sauer, J. B. , Binder, H. , … Mikolajczyk, R. (2014). Quality of life in Parkinson's disease patients with motor fluctuations and dyskinesias in five European countries. Parkinsonism & Related Disorders, 20(9), 969–974. 24953743 10.1016/j.parkreldis.2014.06.001

[brb3837-bib-0013] Hely, M. A. , Reid, W. G. J. , Adena, M. A. , Halliday, G. M. , & Morris, J. G. L. (2008). The Sydney Multicenter Study of Parkinson's disease: The inevitability of dementia at 20 years. Movement Disorders, 23(6), 837–844. 18307261 10.1002/mds.21956

[brb3837-bib-0014] Hoehn, M. M. , & Yahr, M. D. (1967). Parkinsonism: Onset, progression, and mortality. Neurology, 17(5), 427–442. 6067254 10.1212/wnl.17.5.427

[brb3837-bib-0015] Jankovic, J. , & Poewe, W. (2012). Therapies in Parkinsonʼs disease. Current Opinion in Neurology, 25(4), 433–447. 22691758 10.1097/WCO.0b013e3283542fc2

[brb3837-bib-0016] Kaasinen, V. , Kinos, M. , Joutsa, J. , Seppänen, M. , & Noponen, T. (2014). Differences in striatal dopamine transporter density between tremor dominant and non‐tremor Parkinson's disease. European Journal of Nuclear Medicine and Molecular Imaging, 41(10), 1931–1937. 24867256 10.1007/s00259-014-2796-5

[brb3837-bib-0017] Larsen, J. P. , Karlsen, K. , & Tandberg, E. (2000). Clinical problems in non‐fluctuating patients with Parkinson's disease: A community‐based study. Movement Disorders, 15(5), 826–829. 11009186 10.1002/1531-8257(200009)15:5<826::aid-mds1010>3.0.co;2-e

[brb3837-bib-0018] Lees, A. J. , Hardy, J. , & Revesz, T. (2009). Parkinson's disease. Lancet, 373(9680), 2055–2066. 19524782 10.1016/S0140-6736(09)60492-X

[brb3837-bib-0019] Lees, A. J. , Tolosa, E. , & Olanow, C. W. (2015). Four pioneers of L‐dopa treatment: Arvid Carlsson, Oleh Hornykiewicz, George Cotzias, and Melvin Yahr. Movement Disorders, 30(1), 19–36. 25488030 10.1002/mds.26120

[brb3837-bib-0020] Martínez‐Martín, P. , Rodríguez‐Blázquez, C. , Forjaz, M. J. , Alvarez‐Sánchez, M. , Arakaki, T. , Bergareche‐Yarza, A. , … Goetz, C. G. (2014). Relationship between the MDS‐UPDRS domains and the health‐related quality of life of Parkinson's disease patients. European Journal of Neurology, 21(3), 519–524. 24447695 10.1111/ene.12349

[brb3837-bib-0021] Nicoletti, A. , Mostile, G. , Nicoletti, G. , Arabia, G. , Iliceto, G. , Lamberti, P. , … Zappia, M. (2016). Clinical phenotype and risk of levodopa‐induced dyskinesia in Parkinson's disease. Journal of Neurology, 263(5), 888–894. 26964541 10.1007/s00415-016-8075-6

[brb3837-bib-0022] Peto, V. , Jenkinson, C. , Fitzpatrick, R. , & Greenhall, R. (1995). The Development and Validation of a Short Measure of Functioning and Well‐Being for Individuals with Parkinsons‐Disease. Quality of Life Research, 4(3), 241–248. 7613534 10.1007/BF02260863

[brb3837-bib-0023] Schrag, A. , & Quinn, N. (2000). Dyskinesias and motor fluctuations in Parkinson's disease. A community‐based study. Brain, 123, 2297–2305. 11050029 10.1093/brain/123.11.2297

[brb3837-bib-0024] Scott, N. W. , Macleod, A. D. , & Counsell, C. E. (2016). Motor complications in an incident Parkinson's disease cohort. European Journal of Neurology, 23(2), 304–312. 26074125 10.1111/ene.12751

[brb3837-bib-0025] Skorvanek, M. , Rosenberger, J. , Minar, M. , Grofik, M. , Han, V. , Groothoff, J. W. , … van Dijk, J. P. (2015). Relationship between the non‐motor items of the MDS–UPDRS and Quality of Life in patients with Parkinson's disease. Journal of the Neurological Sciences, 353(1–2), 87–91. 25918077 10.1016/j.jns.2015.04.013

[brb3837-bib-0026] Stocchi, F. , Antonini, A. , Barone, P. , Tinazzi, M. , Zappia, M. , Onofrj, M. , … DEEP study group . (2014). Early DEtection of wEaring off in Parkinson disease: The DEEP study. Parkinsonism and Related Disorders, 20(2), 204–211. 24275586 10.1016/j.parkreldis.2013.10.027

[brb3837-bib-0027] Tomlinson, C. L. , Stowe, R. , Patel, S. , Rick, C. , Gray, R. , & Clarke, C. E. (2010). Systematic review of levodopa dose equivalency reporting in Parkinson's disease. Movement Disorders, 25(15), 2649–2653. 21069833 10.1002/mds.23429

[brb3837-bib-0028] Vijayakumar, D. , & Jankovic, J. (2016). Drug‐Induced Dyskinesia, Part 1: Treatment of Levodopa‐Induced Dyskinesia. Drugs, 76(7), 759–777. 27091215 10.1007/s40265-016-0566-3

[brb3837-bib-0029] Warren Olanow, C. , Kieburtz, K. , Rascol, O. , Poewe, W. , Schapira, A. H. , Emre, M. , … Stalevo Reduction in Dyskinesia Evaluation in Parkinson's Disease (STRIDE‐PD) Investigators . (2013). Factors predictive of the development of Levodopa‐induced dyskinesia and wearing‐off in Parkinson's disease. Movement Disorders, 28(8), 1064–1071. 23630119 10.1002/mds.25364

[brb3837-bib-0030] Yoritaka, A. , Shimo, Y. , Takanashi, M. , Fukae, J. , Hatano, T. , Nakahara, T. , … Hattori, N. (2013). Motor and non‐motor symptoms of 1453 patients with Parkinson's disease: Prevalence and risks. Parkinsonism and Related Disorders, 19(8), 725–731. 23639756 10.1016/j.parkreldis.2013.04.001

